# Eccentrically widened bone tunnels after all-inside anterior cruciate ligament reconstruction: a computed tomography and three-dimensional model-based analysis

**DOI:** 10.1007/s00167-022-07164-3

**Published:** 2022-09-22

**Authors:** Di Liu, Zi-Jun Cai, Wen-Hao Lu, Lin-Yuan Pan, Yun-Tao Yang, Yu-Sheng Li, Wen-Feng Xiao

**Affiliations:** 1 Department of Orthopedics, Xiangya Hospital, Central South University, Xiangya Road 87, Changsha, 410008 Hunan China; 2 National Clinical Research Center for Geriatric Disorders, Xiangya Hospital, Central South University, Changsha, 410008 Hunan China

**Keywords:** Anterior cruciate ligament reconstruction, All-inside, Tunnel widening, Tunnel position, Computed tomography

## Abstract

**Purpose:**

To evaluate the extent of tunnel widening after anterior cruciate ligament reconstruction (ACLR) using the all-inside technique and to establish its correlation with patient-reported clinical outcomes and femoral graft bending angle (GBA).

**Methods:**

Tunnel widening was evaluated using computed tomography (CT)-based three-dimensional (3D) models, and the femoral GBA was directly measured on CT images using the Picture Archiving and Communication System (PACS) software. Clinical follow-up was routine procedure, and patient-reported clinical outcomes mainly included International Knee Documentation Committee (IKDC), Lysholm, and Knee Injury and Osteoarthritis Outcome Scores (KOOS) scores, and subjective knee stability assessment.

**Results:**

Fifty-two patients received standard all-inside ACLR, with a median follow-up of 6 months. Reconstructed anterior cruciate ligaments (ACLs) were scanned during the first 3 days and 6 months after surgery. On both the femoral and tibial sides, bone tunnels were most significantly enlarged at the articular aperture segment; the femoral tunnel was 9.2 ± 1.3 mm postoperatively and was significantly enlarged by 32% to a mean tunnel diameter of 12.1 ± 2.0 mm at 6 months after surgery. Moreover, the extent of tunnel enlargement gradually decreased as the measured levels approached those of the bone cortex. The femoral tunnel center was shifted into the anterior and distal direction, and the tibial tunnel center was shifted into the posterior and lateral direction. Additionally, the mean femoral GBA was 105.9° ± 8.1° at the 6-month follow-up. Tunnel enlargement and GBA were not significantly correlated with patient-reported outcomes.

**Conclusions:**

Femoral and tibial tunnels were significantly greater and eccentrically shifted at the 6-month follow-up after all-side ACLR. However, the extent of tunnel widening does not markedly affect the short-term clinical outcomes. Meanwhile, the femoral GBA was not significantly correlated with femoral tunnel widening or patient-reported outcomes. Although the tunnel widening following all-inside ACLR was not associated with clinical outcomes, it potentially caused difficulties in revision ACLR.

**Level of evidence:**

Level IV.

## Introduction

ACL tears are prevalent articular injuries during physical activities, and ACLR is currently the optimal surgery for maintaining the function and stability of knee joints [6, 17, 37, 42]. In 1995, Morgan et al. [34] introduced the initial all-inside technique for ACLR. However, this new technique has since evolved owing to various drawbacks [5, 28]. Currently, the all-inside technique, as described by Lubowitz et al. [28] in 2011, has become an improved and mature surgical method for ACL reconstruction. As the second-generation and no-incision ACLR, the all-inside technique exerts comparable functional outcomes to ACLR using conventional techniques and offers the advantages of improved cosmesis, preserved knee flexor strength, and decreased postoperative pain [5, 21, 27, 28, 36].

Despite the diversity of surgical techniques and development of surgical instruments, ACLR has not reached the acme of perfection and is still accompanied by several problems. One such widely reported issue is bone tunnel widening, which predominantly occurs in the first 6 weeks to 6 months after ACLR [8, 15, 16]. Widened bone tunnels affect graft maturation and bone–tendon healing, and cause difficulties in revision ACLR [14, 18, 32, 45, 51, 53]. However, the specific etiology of tunnel widening remains unclear, with biological and mechanical influences [1, 7, 18, 20, 30]. Synovial fluid-derived osteolytic cytokines, local inflammation response, graft type, and cell necrosis are viewed as important biological factors [43, 52]. The mechanical factors mainly include graft–tunnel micromotion, improper graft position, increased posterior tibial slope, and accelerated rehabilitation [18, 24, 35]. Intriguingly, recent studies have reported that the tunnel enlargement was concomitant with the shift of tunnel positions [24, 46], which may be associated with the uneven mechanical stress distributed on the tunnel walls [19]. With respect to the evaluation of femoral and tibial bone tunnels, CT serves as the most reliable imaging modality compared to plain radiography and magnetic resonance imaging (MRI) [29]. Virtual 3D-CT bone models further increase the accuracy and reliability of the measurement of bone tunnels using imaging analysis software [9].

The femoral GBA refers to the obtuse angle between the femoral axis line and the connecting line of the intra-articular apertures of the femoral and tibial tunnels [48]. Some studies have indicated that an acute GBA negatively affects graft healing and is associated with tunnel widening following the ACLR [26, 41, 48]. However, few studies have investigated the potential role of the femoral GBA in tunnel widening and the clinical outcomes after all-inside ACLR.

The primary purpose of this study was to measure tunnel widening following all-inside ACLR with the assistance of CT scans and 3D-CT bone tunnel models. The correlation between the tunnel enlargement, femoral GBA, and patient-reported clinical outcomes was assessed. It was hypothesized that the all-inside technique would lead to widening of bone tunnels, but that a more acute GBA would not be correlated with tunnel widening, and that the patient-reported clinical outcomes would be satisfactory for patients at the 6-month follow-up.

## Materials and methods

The protocol of this retrospective study was approved by the Institutional Review Board of Xiangya Hospital (No. 202201039), and the written informed consent was obtained from all patients.

## Patients

The patients were recruited between October 2020 and October 2021. All patients who underwent primary ACLR for unilateral ACL rupture using the standard all-inside technique and underwent CT scanning at the 6-month follow-up were included. Patients with meniscal or chondral defects concurrent with ACL rupture were included. The exclusion criteria were as follows: multi-ligament injury, avulsion fracture, revision surgery, re-rupture after primary ACLR, or postoperative infection.

## Surgical technique

All patients underwent a standard all-inside ACLR [5, 28]. All reconstructions were performed by the same experienced orthopedic surgeon. The semitendinosus and/or gracilis tendons were harvested in a minimally invasive fashion and prepared as a multi-stranded graft, which was clamped and sewn with a TightRope adjustable graft loop suspensory cortical button (Arthrex, Naples, FL, USA). Subsequently, the graft was stitched using No. 0 FiberWire high-strength sutures (Arthrex, Naples, FL, USA) on the femoral and tibial sides. Then, this well-prepared graft was tensioned with the final length of ≤ 70 mm. Simultaneously, standard anterolateral (AL) and anteromedial (AM) portals were made, and injuries to the articular ligaments, meniscus, and cartilage were further confirmed under the arthroscopic visualization. A 25-mm femoral socket was drilled at the ACL femoral footprint, and a tibial socket of 30 mm length was drilled in a retrograde manner. Afterward, the autograft was shuttled through the bone tunnels via the AM portal with synchronous retrieval of femoral and tibial graft-passing sutures, before being solidly fixed on the femoral and tibial bony cortex using TightRope button (Arthrex, Naples, FL, USA). Finally, the graft tensioning was performed with full extension.

## Postoperative rehabilitation

Standard postoperative rehabilitation was conducted for all patients who underwent ACLR. Patients were encouraged to begin good quadriceps control from the first day postoperatively. Normally, patients are instructed to attain 90° of flexion of the reconstructed knee within 2 weeks and regain the full range of motion (ROM) in the first month following ACLR. Additionally, tolerable and progressive weight-bearing was allowed as early as possible. An external protection brace with an adjustable angle was recommended to be worn for 3 months postoperatively. Jogging was allowed after 3–6 months, and return to sports activities was started from 12 months postoperatively. It should be noted that the rehabilitation plan was formulated and adjusted based on the patients’ actual state. For example, patients with meniscus repair or suture were advised to delay full weight-bearing and knee bending > 90° until 1 month after surgery.

## Clinical outcome evaluation

Functional evaluations were performed using the subjective IKDC score, Lysholm score, and KOOS score, which were recorded preoperatively and 6 months postoperatively.

## Radiological evaluation and measurement methods

Follow-up CT scans at the initial 3 days and 6-month follow-up after all-inside ACLR were obtained for all patients with full extension of the knees. The standard CT protocol for the lower extremity was mainly performed with following parameters: 120 kV, 200–240 mA, and 0.625-mm or 1.0-mm slice thickness. The field of view covered the entire length of the femoral and tibial tunnels.

**Fig. 1:**
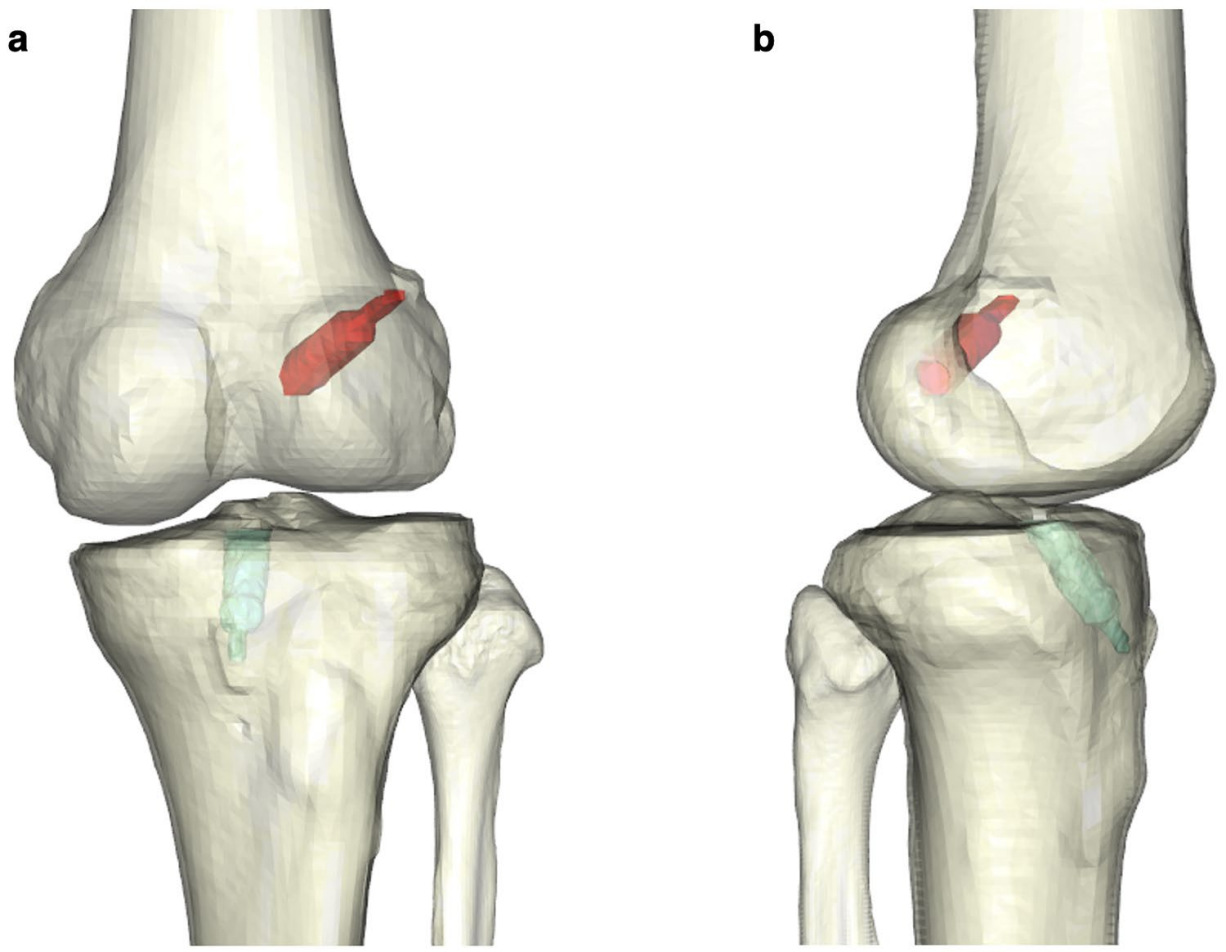
Initial segmented 3D-CT models of femur, tibiofibular, and bone tunnels using the post-processing software (**a** left knee, anterior view; **b** left knee, medial–lateral view)

**Fig. 2:**
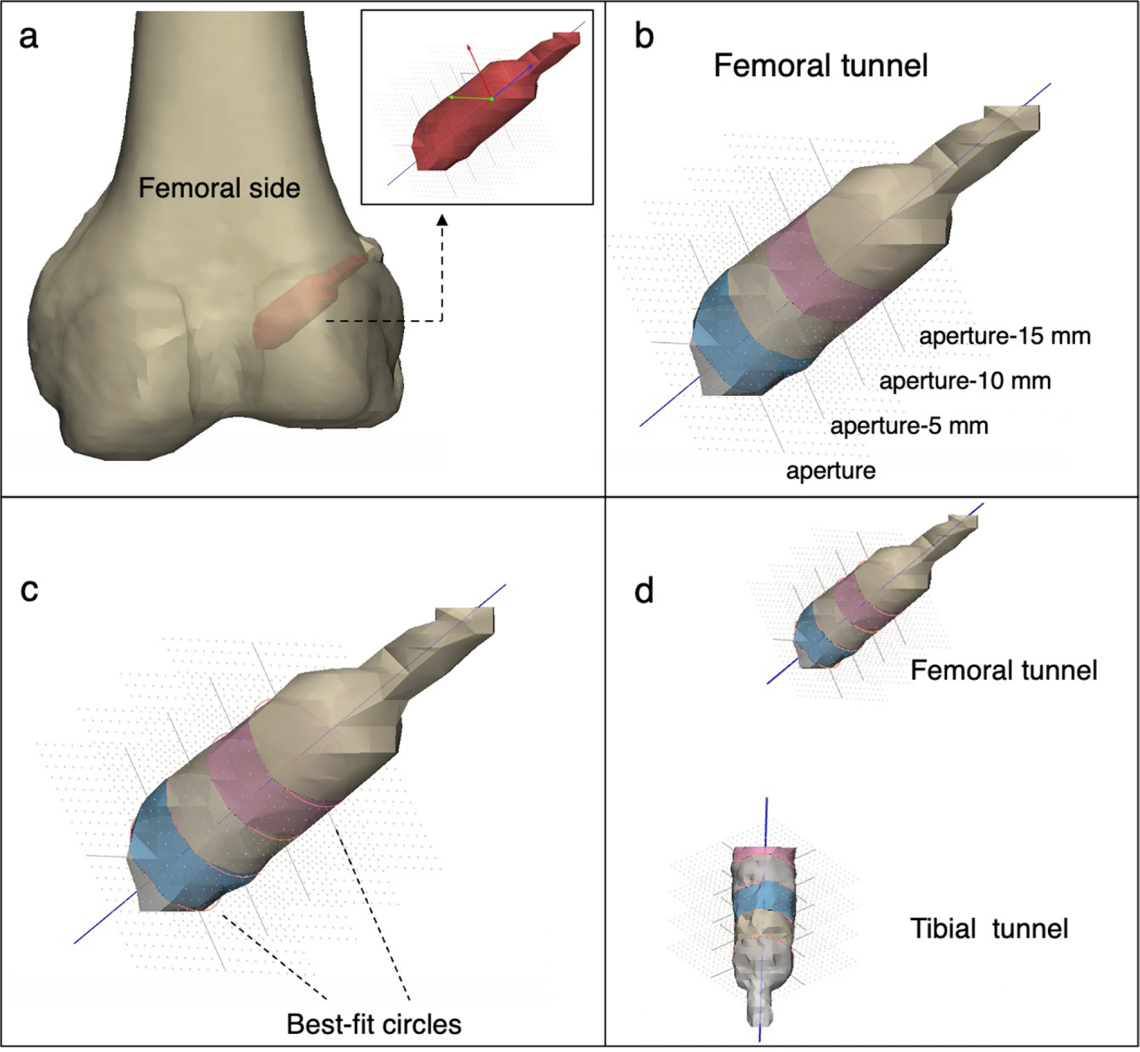
Specific measurement procedures of tunnel diameters. **a** Anterior view, left knee, 3D models of femur and femoral tunnel with a best-fit longitudinal axis and four segments. **b** Anterior view, left knee, a femoral tunnel segmented with a 5-mm interval and four corresponding levels (aperture, aper-5 mm, aper-10 mm, aper-15 mm). **c** Anterior view, left knee, four best-fit circles allowing to measure tunnel diameters. **d** Anterior view, left knee, integral segmented femoral and tibial tunnels

To evaluate tunnel widening, we chose the best-fit transverse section method, which was described in detail by Crespo et al. [9], and regarded it as the most reliable and accurate method for the measurement of bone tunnels after ACLR. In this study, the DICOM data of the CT images were extracted from the PACS software and exported into the image post-processing software Materialise Mimics (version 21.0, Leuven, Belgium). The femurs, tibias, and bone tunnels can be segmented and modeled from other joint structures according to the bone–soft tissue density variation on the CT data (Fig. 1). Then the models of bony structures and bone tunnels were imported into the 3D modeling software Materialise 3-matic (version 13.0, Leuven, Belgium), in which the bone tunnels were visualized and evaluated. According to the best-fit transverse section method, a best-fit center axis was penetrated through the full-length tunnel. On both femoral and tibial sides, the best-fit circles were automatically fitted to the external tunnel walls at four levels with a 5-mm interval: intra-articular aperture, 5 mm from the intra-articular aperture (aper-5 mm), 10 mm from the intra-articular aperture (aper-10 mm), and 15 mm from the intra-articular aperture (aper-15 mm) (Fig. 2). Finally, the diameters of best-fit circles were automatically measured. Notably, these procedures were also manually revised and adjusted in order to ensure the accuracy of measurements.

**Fig. 3:**
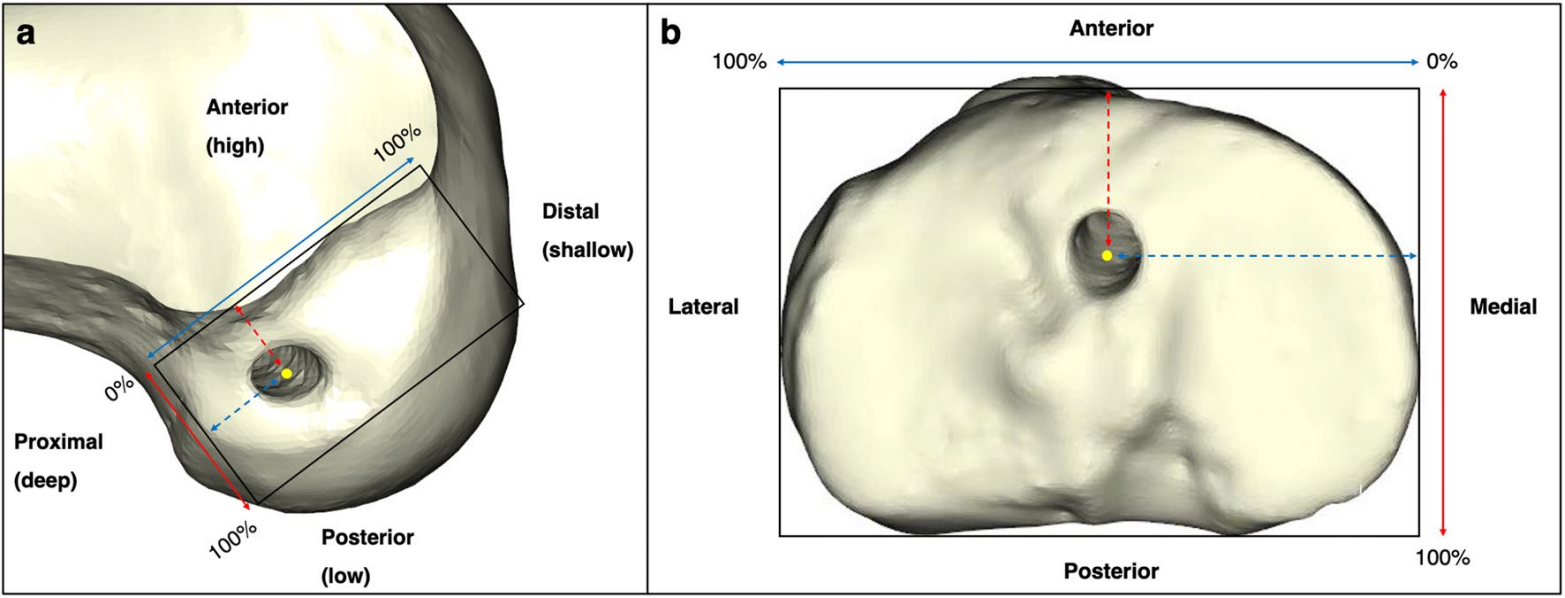
Measurement methods for femoral and tibial tunnel positions. **a** Modified quadrant method for femoral tunnel measurement. The high–low height of the femoral tunnel was measured as a percentage: perpendicular distance from the center of the tunnel to the highest border of the reference frame (dashed red line)/total height of the reference frame (solid red line)*100%. The deep–shallow depth was calculated as a percentage: horizontal distance from the center of the tunnel to the deepest border of the reference frame (dashed blue line)/total depth of the reference frame (solid blue line)*100%. **b** Method for tibial tunnel measurement. The anterior–posterior depth of the tibial tunnel was measured as a percentage: perpendicular distance from the center of the tunnel to the anterior border of the reference frame (dashed red line)/total height of the reference frame (solid red line)*100%. The medial–lateral width was calculated as a percentage: horizontal distance from the center of the tunnel to the medial border of the reference frame (dashed blue line)/total depth of the reference frame (solid blue line)*100%

The tunnel position was assessed based on the 3D femoral and tibial models of the femurs and tibias (Materialise Mimics 21.0 and Materialise 3-matic 13.0, Leuven, Belgium). The femoral model was placed horizontally parallel in the strict lateral position, ensuring the both femoral condyles were virtually overlapped. Subsequently, the model was rotated to the neural position in the distal view and the medial femoral condyle was removed using a perpendicular surface that contains the highest point of the anterior aperture of the intercondylar notch as the reference. Finally, the femoral model was rotated to the strict lateral view. For the tibial model, it was rotated to the posterior view and tibial condyles were horizontally parallel. Then the tibial model was rotated about 90° to the top view of tibial plateau, in which the posterior articular margins of medial and lateral condyles were placed in the same horizontal level. Snapshots of the medial–lateral view of the lateral femoral condyle and the top view of the tibial plateau were recorded and measured. The methods of measurement to assess the shifting orientation of femoral and tibial tunnels were fully described previous studies [3, 24, 25, 33]. The femoral tunnel positions were measured by the modified quadrant method purposed by Edwards et al. [3, 13, 25], in which a rectangular reference frame was drawn with the superior border of intercondylar notch roof, and the lowest, shallowest, and deepest lateral walls of the intercondylar notch. For the tibial tunnel position, the cortical outline of the proximal tibia was used as the borders of a rectangular reference frame for the measurement [25, 33] (Fig. 3). The post-processing and remodeling procedures were performed by a well-trained independent orthopedic investigator who was not involved in the surgery or patient care.

**Fig. 4:**
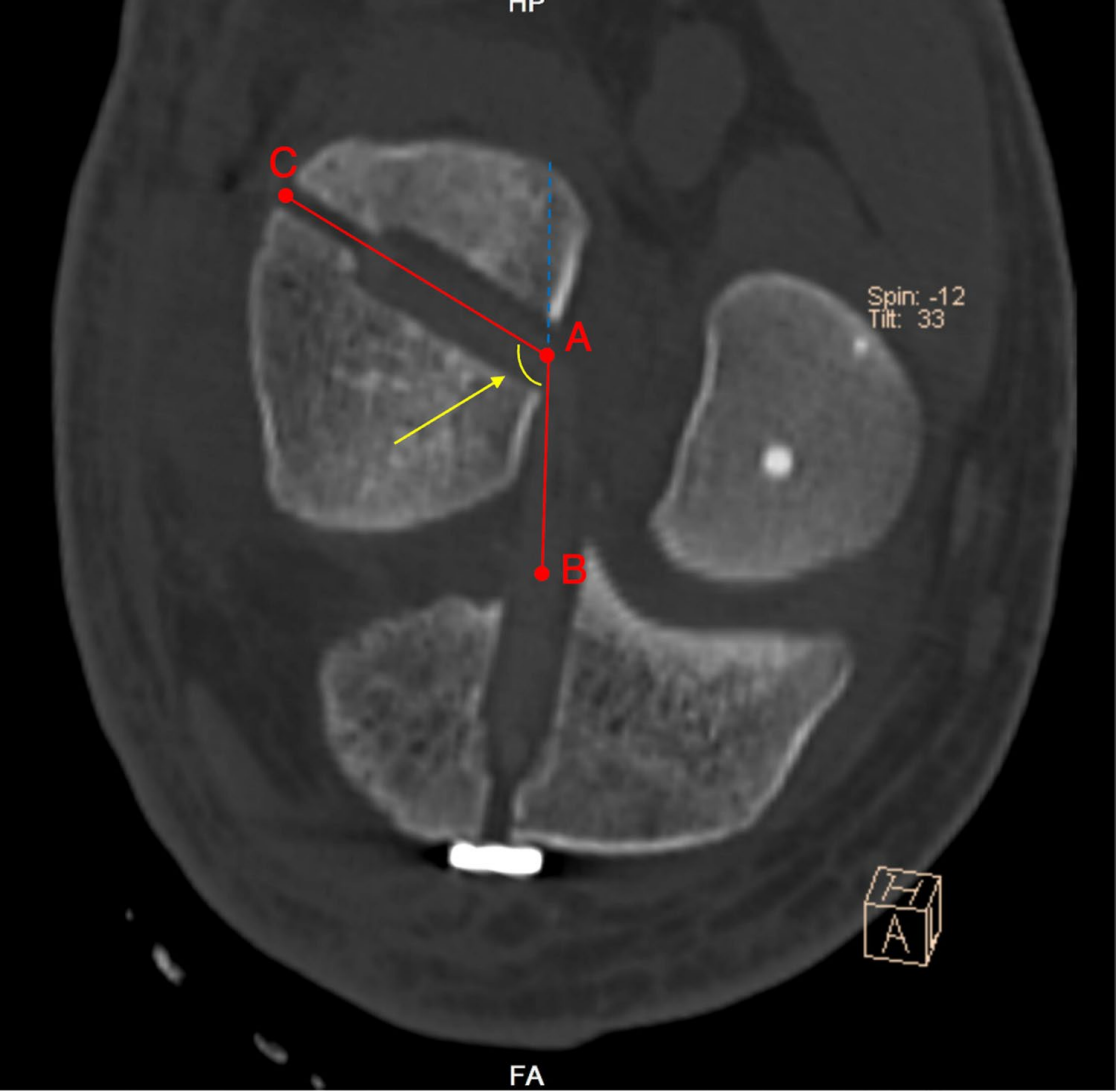
Measurement of the femoral GBA (yellow arrow). ∠CAB, the femoral GBA; AB, the line connecting the intra-articular apertures of the femoral and tibial tunnels; AC, the line connecting the extra- and intra-articular apertures of the femoral tunnel

The femoral GBA plane that contains the longitudinal axes of both femoral and tibial tunnels was post-processed on the workstation (Syngo, VB20A, Siemens Healthineers) using the multi-planar reconstruction (MRP) mode. The femoral GBA was evaluated using the PACS software by measuring the obtuse angle between the central axis of the femoral bone tunnel and the line connecting the articular apertures of the femoral and tibial tunnels, as previously proposed and described by Wang et al. [48] (Fig. 4).

All of the aforementioned diameters and radiographic findings were independently measured and evaluated by two experienced orthopedic surgeons.

## Statistical analysis

Descriptive statistics were used to describe the patients’ general characteristics. Statistical analysis was performed using SPSS software (version 26.0, IBM Corp., Armonk, NY, USA). The Wilcoxon signed-rank test or paired *t*-test was adopted to compare the patient-reported outcomes, tunnel diameters, and tunnel positions respectively. The Pearson’s correlation and Spearman’s rank correlation were chosen to analyze the correlation between tunnel diameter, femoral GBA, clinical outcomes, and other potential influencing factors. The intra-class correlation coefficient (ICC) was performed to evaluate the intra- and interobserver agreements for the measurements of imaging findings using the two-way random model. Statistical significance was set at *P* < 0.05. To detect the differences of tunnel diameters with 90% power using the paired *t*-test at a significance level of 0.05, a sample size of 14 was required based on published data from Monaco et al. [32]. The sample size was performed using PASS software (version 15.0.5, NCSS, Kaysville, Utah, USA).

## Results

### Main demographics

The patient demographics were summarized in Table 1. Fifty-two patients (38 males and 14 females) who underwent all-inside ACLR were enrolled in this study. The mean patient age was 26.4 ± 8.2 years (range 14–48 years), and the mean body mass index (BMI) was 24.4 ± 3.9. The median time from surgery to follow-up was 185.5 days (range 167–232 days).

**Table 1:** Main demographics of patients

Age, y ^*^	26.4 ± 8.2 (14–48)
Sex, male/female	38/14
BMI, kg/m^2 *^	24.4 ± 3.9 (15.9–32.9)
Time from surgery to follow-up, days ^#^	185.5 (167–232)
Torn side of ACL, left/right	29/23
Meniscal injury, *n* (%)	38 (73.1%)
Medial/lateral ^a^	24/25
Plasty/meniscectomy/suture ^b^	8/3/39
Chondral injury, *n* (%)	16 (30.8%)
Autograft type, ST/ST + G	40/12

*BMI* body mass index, *n* number, *ST* semitendinosus, *G* gracilis

^a^Some patients suffered both medial and lateral meniscus tear

^b^One medial meniscus was treated by meniscectomy and suture

*Data expressed as mean ± SD (range values)

^#^Data expressed as median (range values)

### Patient-reported outcomes

All patient-reported clinical outcomes were significantly improved compared to the preoperative conditions (*P* < 0.05). All patients returned to varying degrees of sporting activities, did not require revision surgery, and suffered no incidences of joint infection, subjective knee instability, or other serious adverse events during the follow-up period (Table 2).

**Table 2:** Clinical outcomes

	Postoperative, 3 days	Postoperative, 6 months	*P* value
IKDC ^*^	46.2 ± 21.8 (2.3–90.8)	73.7 ± 12.7 (44.8–100.0)	** < *0.05***
Lysholm ^#^	65.5 (48.3–77.0)	85.0 (69.0–90.0)	** < *0.05***
KOOS			
Symptoms ^#^	71.4 (50.0–82.1)	89.3 (78.6–92.9)	** < *0.05***
Pain ^#^	77.8 (63.9–83.3)	91.7 (83.3–99.3)	** < *0.05***
ADL ^#^	80.1 (64.7–93.8)	97.1 (91.2–100.0)	** < *0.05***
Sport ^#^	35.0 (11.3–70.0)	75.0 (60.0–85.0)	** < *0.05***
QoL ^*^	43.8 ± 25.0 (0.0–93.8)	64.2 ± 18.0 (18.8–100.0)	** < *0.05***

A *P* value of < 0.05 indicates statistical significance and was highlighted in bold italics

*ADL* Activities of daily living subscale; *Sport* Sport and recreation subscale; *QoL* Quality of life subscale

*Data expressed as mean ± SD (range values)

^#^Data expressed as median *P*_25_–*P*_75_

### Change in tunnel diameters and positions

Over the 6-month follow-up, the femoral and tibial tunnels were enlarged following all-inside ACLR, and the degree of femoral tunnel enlargement was wider than that of the tibial side at every measured level. On both the femoral and tibial sides, the bone tunnels were primarily widened at the articular aperture segments (*P* < 0.05). On the femoral side, the tunnel diameter was significantly enlarged by 32% to a mean tunnel diameter of 12.1 ± 2.0 mm at the section of articular aperture (*P* < 0.05); on the tibial side, the tunnel diameter was increased by 15% to 10.8 ± 1.3 mm at the articular level (*P* < 0.05). Additionally, the degree of tunnel enlargement gradually decreased as the measured levels approached those of the cortical bone (Table 3).

**Table 3:** Tunnel diameter in the first 3 days and 6 months after ACLR

	Postoperative, 3 days	Postoperative, 6 months	Δ Tunnel diameter	*P* value
Femoral tunnel				
Aperture	9.2 ± 1.3	12.1 ± 2.0	2.9 ± 1.6	** < *0.05***
Aper-5 mm	9.5 ± 1.6	11.5 ± 2.3	2.0 ± 1.9	** < *0.05***
Aper-10 mm	9.4 ± 1.6	10.4 ± 2.2	1.1 ± 1.7	** < *0.05***
Aper-15 mm	9.2 ± 1.8	8.8 ± 2.3	− 0.4 ± 1.7	n.s
Tibial tunnel				
Aperture	9.4 ± 1.4	10.8 ± 1.3	1.3 ± 1.4	** < *0.05***
Aper-5 mm	9.2 ± 1.5	10.3 ± 1.4	1.1 ± 1.6	** < *0.05***
Aper-10 mm	9.0 ± 1.5	9.5 ± 1.4	0.4 ± 1.7	n.s
Aper-15 mm	8.6 ± 1.6	8.0 ± 1.8	− 0.6 ± 2.0	** < *0.05***

Data expressed as mean ± standard deviation (SD)

A *P* value of < 0.05 indicates statistical significance and was highlighted in bold italic

Aper-5/10/15 mm indicates the sections at 5 or 10 or 15 mm from the articular aperture, respectively

Δ indicates the tunnel diameter difference between the first 3 days and 6 months postoperatively

The center of the femoral tunnels was eccentrically shifted from 50.0 ± 6.2% to 46.3 ± 7.1% in the anterior–posterior (high–low) direction (*P* < 0.05) and from 26.9 ± 3.5% to 32.3 ± 5.0% in the proximal–distal (deep–shallow) direction (*P* < 0.05). The center of the tibial tunnels was migrated from 36.8 ± 3.4% to 38.6 ± 4.4% in the anterior–posterior direction (*P* < 0.05) and from 46.2 ± 2.4% to 47.2 ± 2.1% in the medial–lateral direction (*P* < 0.05) (Table 4).

**Table 4:** Tunnel position in the first 3 days and 6 months after ACLR

	Postoperative, 3 days	Postoperative, 6 months	Δ Tunnel position	*P* value
Femoral tunnel				
Anterior–posterior (high–low) (%)	50.0 ± 6.2	46.3 ± 7.1	− 3.7 ± 5.4	** < *0.05***
Proximal–distal (deep–shallow) (%)	26.9 ± 3.5	32.3 ± 5.0	5.3 ± 3.6	** < *0.05***
Tibial tunnel				
Anterior–posterior (%)	36.8 ± 3.4	38.6 ± 4.4	1.7 ± 2.7	** < *0.05***
Medial–lateral (%)	46.2 ± 2.4	47.2 ± 2.1	0.9 + 1.7	** < *0.05***

Data expressed as mean ± standard deviation (SD)

A *P* value of < 0.05 indicates statistical significance and was highlighted in bold italic

Δ indicates the tunnel position difference between the first 3 days and 6 months postoperatively

The intra- and interobserver measurements exerted an almost excellent reliability (Table 5).

**Table 5:** Intra- and inter-reliability on the measurements of tunnel diameters and positions

	Tunnel widening		Tunnel position	
	ICC	95% CI	ICC	95% CI
Intra-observer				
Observer 1	0.96	0.94–0.98	0.91	0.86–0.95
Observer 2	0.93	0.90–0.96	0.87	0.81–0.92
Inter-observer	0.94	0.92–0.96	0.88	0.82–0.93

*ICC* Intra-class correlation coefficient, *95*% *CI* 95% confidence interval

### Correlation between tunnel widening and clinical outcomes

Although the tunnel diameters were significantly increased on both the femoral and tibial sides, the tunnel diameter differences (Δ) were not correlated with the patient-reported outcomes (n.s) (Table 6).

**Table 6:** Correlation of the tunnel diameter difference (Δ) on both of femoral and tibial sides with clinical outcomes

	Δ Femoral aperture		Δ Femoral aper-5 mm		Δ Tibial aperture		Δ Tibial aper-5 mm	
	*r*/*r*_*s*_	*P* value	*r*/*r*_*s*_	*P* value	*r*/*r*_*s*_	*P* value	*r*/*r*_*s*_	*P* value
IKDC ^#^	− 0.07	n.s	− 0.04	n.s	− 0.09	n.s	− 0.10	n.s
Lysholm ^*^	− 0.12	n.s	− 0.17	n.s	− 0.13	n.s	− 0.15	n.s
KOOS								
Symptoms ^*^	− 0.02	n.s	− 0.09	n.s	− 0.05	n.s	− 0.16	n.s
Pain ^*^	0.10	n.s	0.08	n.s	0.09	n.s	0.07	n.s
ADL ^*^	0.06	n.s	0.02	n.s	0.08	n.s	0.06	n.s
Sport ^*^	0.11	n.s	− 0.03	n.s	0.08	n.s	0.06	n.s
QoL ^#^	0.16	n.s	0.20	n.s	0.01	n.s	0.02	n.s

A *P* value of < 0.05 indicates significant statistical correlation and was highlighted in bold italics

Δ indicates the tunnel diameter difference between the first 3 days and 6 months postoperatively

*r*/*r*_*s*_ correlation coefficient

*Data analyzed by the Spearman rank correlation

^#^Data analyzed by the Pearson correlation

### Correlation of femoral GBA with tunnel widening and clinical outcomes

The mean femoral GBA was 105.9° ± 8.1° (89.0°−130.8°). The femoral GBA did not correlate with size differences in the femoral tunnel (n.s) and exerted poor correlation with primary clinical outcomes (Table 7).

**Table 7:** Correlation of GBA with tunnel widening and clinical outcomes

	Femoral graft bending angle (°)	
	*r*/*r*_*s*_	*P* value
Δ Femoral aperture ^#^	0.11	n.s
Δ Femoral aper-5 mm ^#^	0.16	n.s
IKDC ^#^	− 0.16	n.s
Lysholm ^*^	− 0.37	** < *0.05***
KOOS		
Symptoms ^*^	− 0.19	n.s
Pain ^*^	− 0.32	** < *0.05***
ADL ^*^	− 0.20	n.s
Sport ^*^	− 0.28	** < *0.05***
QoL ^#^	− 0.18	n.s

A *P* value of < 0.05 indicates significant statistical correlation and was highlighted in bold italics

*r*/*r*_*s*_ correlation coefficient

*Data analyzed by the Spearman rank correlation

^#^Data analyzed by the Pearson correlation

### Analysis of influencing factors on tunnel widening

BMI was statistically correlated with increased diameters of the aper-5 mm of the femoral tunnel (*P* < 0.05, *r* = 0.32) and the aperture of the tibial tunnel (*P* < 0.05, *r* = 0.28). The time from surgery to the eventual follow-up and age were not significantly correlated with the tunnel enlargement (n.s).

## Discussion

The most important finding of the present study was that both of the femoral and tibial bone tunnels were eccentrically widened following ACLR with an all-inside technique. However, the degree of enlargement was not correlated with short-term patient-reported outcomes. Additionally, the femoral GBA did not correlate with the femoral tunnel diameter and clinical outcomes.

The occurrence of tunnel widening following ACLR is a prevalent phenomenon and has been widely reported [2, 50]. Most cases of bone tunnel enlargement arose during the first 6 weeks or 6 months following ACLR; however, the size of the widened tunnels gradually decreased 1 year after surgery and no further increase in tunnel size was observed 2–3 years later [12, 15, 16, 30, 31]. Thus, CT images taken 6 months after all-inside ACLR were used to evaluate the degree of tunnel widening. CT scanning has the highest reliability compared to plain radiography and MRI, and 3D-CT-based tunnel models have provided an intuitive approach to evaluate the size of bone tunnels [9, 29, 32, 38]. Despite the effectiveness of this measuring tool, the specific mechanisms of tunnel enlargement have not yet been fully elaborated, and multifactorial causes are known to contribute to the greater tunnel diameters. Indeed, recent studies have demonstrated that both biological and mechanical factors are involved in the occurrence and progression of tunnel widening after ACLR. Biological factors include infiltration of osteolytic cytokines into the bone–graft space, nonspecific inflammatory responses, and cell necrosis; mechanical factors include graft–tunnel micromotion, improper graft placement, and aggressive rehabilitation [1, 7, 18, 20, 30]. Notably, it is inevitable that the reconstructed autograft will move slightly in the graft–tunnel space along the transverse and longitudinal directions. The greater tunnel widening was occurred on the femoral side, which is in line with the findings of previous studies [30, 32]. From a mechanical viewpoint, there is likely to be less relative movement between the autograft and tibial tunnel compared to that on femoral side. Moreover, the results of this study showed that the tunnel diameters were primarily enlarged at the articular aperture level in comparison with tunnel segments closer to the cortical bones. This phenomenon was partially attributed to larger transverse motion of autografts and longer direct immersion of adverse bioactive factors from synovial fluid at the intra-articular aperture level [22]. Although postoperative tunnel enlargement has been extensively documented, widened bone tunnels did not correlate with inferior patient-reported outcomes. In this study, the patient-reported outcomes were significantly improved after all-inside ACLR. However, tunnel widening did not correlate with any of the clinical outcome scores. These results demonstrate that although tunnel widening is a prevalent imaging finding, its clinical relevance remains unclear [39, 44, 49]. Intriguingly, the present results indicated that the tunnel enlargement produced an eccentrical shift of the femoral tunnel position in the anterior and distal direction, and the tibial tunnel position was slightly shifted into the lateral and posterior direction. Accordingly, the mechanical traction force of the autograft constantly produces eccentric stress on tunnel walls and thus contributes to tunnel widening in the direction where the graft runs and pulls [19, 24, 30]. The eccentrical tunnel shifting may adversely affect the bone–tendon healing due to less contact area between the autografts and tunnel walls and cause difficulties for revision ACLR.

In our patients, the mean femoral GBA has been measured as 105.9° ± 8.1° and it has not been significantly correlated with the tunnel enlargement. Although a greater GBA was correlated with some of worse patient-reported outcomes in our study, these correlations were too weak to verify that the GBA affects clinical outcomes. In a previous study, Lee et al. [23] also demonstrated that the GBA did not significantly affect the enlargement of bone tunnels and clinical outcomes, regardless of the surgical techniques. However, several other studies found that an acute GBA contributed to the occurrence of tunnel widening, but did not negatively affect the clinical outcomes [26, 47]. Theoretically, a high or acute GBA increases the stress on the graft–tunnel interface during physical activities; however, in practice, the causal role of the GBA in bone tunnels and clinical outcomes after all-inside ACLR requires further investigated.

BMI was poorly correlated with only two enlarged segments of bone tunnels (femoral aper-5 mm and tibial aperture). Additionally, the time from surgery to follow-up and age were not correlated with the enlarged diameters on either of the femoral and tibial sides. Accordingly, our results do not support that BMI, the time from surgery to follow-up, and age, as independent risk factors, contribute to the tunnel widening after all-inside ACLR.

The results indicated that the tunnel widening has occurred after all-inside ACLR, but that the extent of eccentric tunnel widening had no significant impact on the clinical outcomes. Several studies have reached the consensus that tunnel widening does not compromise clinical outcomes following ACLR, at least in the short- and intermediate term, although larger bone tunnels may be gradually stabilized or even decreased over time with bone–tendon healing [7, 31, 32, 45]. Therefore, all-inside ACLR exhibits good surgical treatment effects, patient-reported outcomes, postoperative stability, and low failure rates equivalent to conventional techniques, and has minimal invasion, improved cosmesis, better pain control, and superior knee flexor strength [4, 10, 11, 21, 32, 40].

This study has some limitations. Firstly, as a retrospective study design is inherently accompanied by selection bias and confounding factors, which may affect radiographic and patient-reported outcomes. Thus, strict exclusive and inclusive criteria were formulated for this study. Besides, the number of enrolled patients was relatively small and the follow-up period was short. The limited follow-up period cannot reflect the final clinical outcomes, and the bone tunnels progressively enlarge over time. However, previous studies have demonstrated that the most obvious tunnel widening occurs in the first 6 weeks following ACLR and decreased slightly after 1 year. And a larger sample size was required to detect the stronger power for the correlation between tunnel diameters and clinical outcomes. Moreover, the quality of the CT images was relatively variable in this study, as more than three high-resolution CT scanners were available in the hospital. However, all of the patients were scanned using radiographic protocols for the lower extremity. A slice thickness of ≤ 1.0 mm and a high resolution were set to ensure the accuracy and reliability of tunnel remodeling. Additionally, it may be more reasonable to compare tunnel widening with other groups based on the surgical approach or graft type.

## Conclusions

With the reliable measurement of 3D-CT analysis, both the femoral and tibial tunnels were eccentrically enlarged at a median follow-up of 6 months following ACL reconstruction using the all-inside technique. Nevertheless, the extent of tunnel widening did not markedly affect the patient-reported clinical outcomes in the short term after all-inside ACLR. Moreover, the femoral GBA was not significantly correlated with tunnel widening or clinical outcomes.

## Abbreviations

ACL: Anterior cruciate ligament
GBA: Graft bending angle
CT: Computed tomography
3D: Three-dimensional
ACLR: Anterior cruciate ligament reconstruction
MRI: Magnetic resonance imaging
ROM: Range of motion
IKDC: International knee documentation committee
KOOS: Knee injury and osteoarthritis outcome scores
PACS: Picture archiving communication system
BMI: Body mass index
ICC: Intra-class correlation coefficient
95% CI: 95% Confidence interval
